# The Effect of Non-Thermal Processing on the Fate of Pathogenic Bacteria and Hidden Hazardous Risks

**DOI:** 10.3390/foods14132374

**Published:** 2025-07-04

**Authors:** Yanan Wu, Xinxin Li, Xinyu Ma, Qing Ren, Zhanbin Sun, Hanxu Pan

**Affiliations:** School of Light Industry Science and Engineering, Beijing Technology and Business University, Beijing 100048, China; wyn2769198642@163.com (Y.W.); lxx199999@126.com (X.L.); mxy15692280959@126.com (X.M.); renqing@th.btbu.edu.cn (Q.R.); twins5616@126.com (Z.S.)

**Keywords:** non-thermal processing, microbial inactivation, viable but nonculturable state, hazardous risks

## Abstract

Non-thermal processing encompasses a range of emerging food technologies, including high-pressure processing (HPP), pulsed electric field (PEF), cold atmospheric plasma (CAP), high-pressure carbon dioxide (HPCD), and ultrasound (US). Unlike traditional thermal processing or chemical preservatives, these methods offer advantages such as lower energy consumption, enhanced environmental sustainability, and effective microbial inactivation, thereby extending food shelf life. Moreover, they can better preserve the nutritional integrity, color, flavor, and texture of food products. However, a critical concern associated with non-thermal processing is its potential to induce microorganisms into a viable but nonculturable (VBNC) state. These VBNC cells evade detection via conventional culturing techniques and may remain metabolically active and retain virulence, posing hidden food safety risks. Despite these implications, comprehensive reviews addressing the induction of a VBNC state by non-thermal treatments remain limited. This review systematically summarizes the microbial inactivation effects and mechanisms of non-thermal processing techniques, the VBNC state, and their associated hazards. This review aims to support technological innovation and sustainable advancement in non-thermal food processing.

## 1. Introduction

Traditional food preservation techniques primarily rely on chemical methods (e.g., preservatives like benzoic acid and sorbic acid or sanitizers like ethanol, sodium hypochlorite, and hydrogen peroxide) and thermal processing (e.g., pasteurization and ultra-high temperature) to prevent or delay microbial growth. However, the disinfectants used in chemical pasteurization may pose residue risks that affect both human health and product quality, and excessive or prolonged antibiotic usage leads to the development of bacterial persistence [1,2]. While thermal pasteurization methods are well-established and widely used for microbial inactivation, they also degrade heat-sensitive nutrients such as vitamin C, polyphenols, and carotenoids in processed products. They also cause unfavorable modifications to sensory qualities (flavor, color, and texture) and may produce potentially harmful byproducts such as acrylamide [3,4,5,6]. In recent decades, growing consumer demand for food safety, food quality, and the retention of nutritional value has driven the development of non-thermal processing technologies.

Unlike traditional heat-based methods, non-thermal food processing uses physical or biological techniques to process food. The differences between thermal and non-thermal processing have been compared and are shown in Table 1. These technologies inactivate microorganisms and enzymes while preserving the original texture, flavor, and nutrients of food, with low energy consumption and minimal environmental pollution [7]. Common non-thermal processing methods include high-pressure processing (HPP), pulsed electric fields (PEFs), ultrasonic (US) processing, high-pressure carbon dioxide (HPCD), cold atmospheric gas plasma (CAP), and photosensitization [7]. The continuous development of technology has led to broader applications of non-thermal processing in diverse food categories including fruits and vegetables, liquid food, ready-to-eat meat and egg products, with the technology now gradually transitioning toward large-scale production.

**Table 1:** Comparison between thermal processing and non-thermal processing.

	Food Sample	Thermal Processing	Non-Thermal Processing	Reference
Energy consuming	/	/	PEF: the natural gas savings were estimated at 100%, electricity savings can be up to 18%; HPP: specific energy input required for sterilization of cans can be reduced from 300 to 270 kJ/kg	[8]
Phenolic compounds-anthocyanins	Aronia berry juice	1.21 ± 0.01 mg/mL	1.55 ± 0.02 mg/mL (HPP)	[9]
Microbial inactivation-total aerobic plate counts		3.86 ± 0.19 mg/mL	3.50 ± 0.34 mg/mL (HPP)	[9]
Environment friendly	/	/	HPCD: utilization of greenhouse gas, CO_2_; HPP: the treatment process is waste-free	[8]
Volatile compounds	Orange juice	22.4% of ethyl butyrate was lost; hexanal and hexyl acetate were virtually lost	PEF: 5.1% of ethyl butyrate were lost; hexanal and hexyl acetate were lost by 7% and 8.4%	[10]
Nutrient value	Lettuce juice	Vc: 1.25 ± 0.02 μg/100 g	Vc: 0.22 ± 0.01 μg/100 g	[5]
		Total chlorophyll: 51.91 ± 0.60 μg/mL	Total chlorophyll: 61.18 ± 1.61 μg/mL	[5]
Sensory quality	Quince Juice	Higher color value	HPCD: Lower color value	[11]
	Lettuce juice	Browning index: 40.95 ± 7.67	Browning index: 27.74 ± 3.12	[5]
	/	Generation of acrylamide	/	[6]

/: not applicable.

In non-thermal food preservation technologies, microbial viability is a pivotal factor determining treatment efficacy and food safety outcomes. The induction of sublethal stress responses in foodborne pathogens through these processing methods may alter their subsequent survival and proliferation characteristics [12]. Non-thermal processing technologies undoubtedly exhibit effective microbial inactivation. However, they may also introduce hidden risks, primarily due to the induction of a viable but nonculturable (VBNC) state in microorganisms [13]. In this state, microbes remain alive and capable of resuscitation under favorable conditions yet evade detection via conventional culture-based methods [14]. Sporulation is a well-known mechanism, but the VBNC state represents another critical survival strategy, particularly for non-spore-forming bacteria [14]. In the VBNC state, bacteria often exhibit a dwarfed cell morphology, significantly reduced metabolic activity, and enhanced resistance to environmental stresses. Morphological changes are closely related to changes in cell wall components, such as peptidoglycan cross linking, lipoprotein, and glycan strands, which result in greater resistance to external stresses [13]. Moreover, cells that appear unculturable might retain the metabolic functions and pathogenic characteristics of viable organisms [15,16]. For example, certain VBNC pathogens, including select *Vibrio* species and *Legionella pneumophila*, maintained their pathogenic potential by continuing to produce toxins [17,18]. This retention of virulence was also confirmed by studies demonstrating that VBNC *Campylobacter jejuni* invaded human intestinal epithelial cells, while VBNC *Listeria monocytogenes* reduced the lifespan of *Caenorhabditis elegans* [19,20]. Most importantly, a series of food-poisoning incidents in Japan caused by VBNC enterohemorrhagic *Escherichia coli*-contaminated salted salmon roe was reported [14], indicating an actual public health risk. Therefore, addressing VBNC issues, elucidating the underlying mechanisms of VBNC formation, and optimizing the intensity of non-thermal processing are critical to ensuring the safety of non-thermally processed food products.

Although numerous studies have investigated the profound microbial effects of non-thermal technologies, the hidden risks of VBNC cells cannot be neglected. To date, studies on VBNC induction using non-thermal treatments and internal mechanisms are emerging, which challenge the extensive application of these techniques. Therefore, exploring both sides of these techniques is important for their further development and application. However, comprehensive reviews on the subject, especially on the VBNC state induced by non-thermal treatments and the differences in the VBNCs induced by various technologies, remain scarce. Therefore, this review summarizes microbial inactivation effects, the inactivation mechanisms of common non-thermal processing technologies, the induction of the VBNC state by these technologies, hazardous risks, and relative formation mechanisms, aiming to provide references and insights for the innovation and sustainable development of non-thermal processing technologies.

## 2. Microbial Inactivation Using High-Pressure Processing (HPP) Technology and Potential Hazardous Risks

### 2.1. Microbial Inactivation via HPP

HPP is one of the most common non-thermal technologies used in the food industry. HPP operates by exerting pressure on food products through a pressurized fluid (typically water) within a high-resistance steel vessel. Initially, a high-flow, low-pressure pump is utilized to fill the container with water. Subsequently, a special pump known as a pressure intensifier is employed to increase the water pressure to the required level [21]. The pressure, which depends on the food product type, is generally maintained within a range of 100–800 MPa [22]. Compared to thermal processing, HPP can achieve bacterial inactivation at ambient temperature, effectively preventing the occurrence of the Maillard and caramelization reactions that happen during thermal processing [23]. The high pressure only affects non-covalent bonds, such as hydrogen bonds, ionic bonds, and hydrophobic bonds, causing changes in the physical and chemical properties and activities of the biological macromolecules in food, such as protein denaturation and enzyme inactivation, thereby achieving microbial inactivation [24,25]. The bacterial inactivation effects of HPP in various types of foods are summarized in Table 2. HPP technology is widely used in various food-processing applications, including the pasteurization of fruit and vegetable beverages, dairy products, and meat products and shell removal for seafood, such as shellfish and shrimp. This technology offers the advantage of allowing pasteurization of packaged products. However, HPP is particularly effective for liquid or semi-solid foods with high water activity, such as juices, beverages, dairy products, and fruit purees, as the aqueous environment enhances microbial inactivation [22]. Although the composition of foods such as juice, meat, and dairy products is complex, the microbial inactivation effects of HPP remain significant. Notably, when HPP was combined with supplementary methods such as freezing or the addition of nisin, the inactivation efficacy was further enhanced. However, although HPP is used commercially, the high equipment and maintenance costs significantly limit the extensive application of this technology.

### 2.2. Bactericidal Mechanisms of HPP

**Figure 1:**
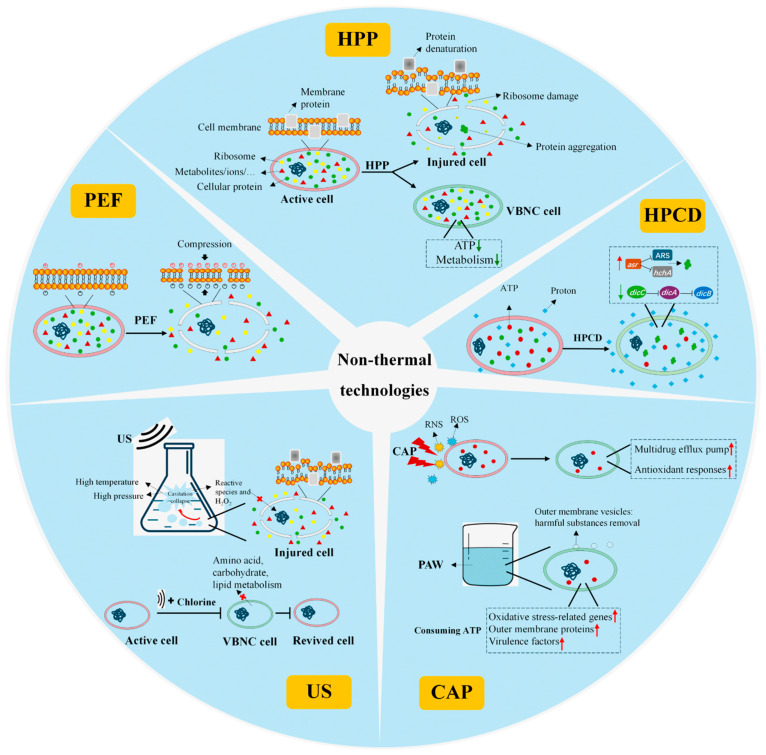
Bacterial inactivation mechanisms or VBNC state formation mechanisms upon the treatment of typical non-thermal processing technologies. HPP treatment: HPP induced phase transitions in the lipid bilayer, reduced membrane protein content, and disrupted cell membrane integrity. These changes increased cellular permeability and promoted the release of intracellular components. Furthermore, intracellular ribosomes represented another critical target of HPP. The treatment altered the higher-order structure of proteins, leading to destabilization and subsequent aggregation of intracellular proteins. PEF treatment: PEF pasteurization generated transmembrane potential, which further caused irreversible electroporation of the cell membrane. These changes caused the leakage of cellular contents to the outside of the cell, which disrupted the balance of ion concentrations inside and outside the cell membrane, affected cellular metabolic activity, and ultimately resulted in cell inactivation. CAP treatment: RNS and ROS generated from CAP treatment induced the formation of the VBNC state, which was related to the mechanisms of cellular energy depletion, antioxidant responses, and upregulation of the multidrug efflux pump. While in PAW-induced VBNC cells, secretion of outer membrane vesicles was contributory to the removal of harmful substances. Additionally, the expression of oxidative stress-related genes, outer membrane proteins, and virulence factors resulted in the severe depletion of intracellular ATP levels, which might serve as a key driver for the VBNC state. HPCD treatment: The high expression of *asr* suppressed acid resistance systems and *hchA* (protein stabilization factor) expression, leading to endogenous protein aggregation and VBNC state formation. Additionally, the gene of *dicC* was downregulated, which could suppress VBNC state formation, while *dicA* promotes entry into the VBNC state through increasing *dicB* expression. Therefore, cell division was ceased and the VBNC state was formed. US treatment: The cavitation effect involved bubble formation, growth, and collapse. This collapse generates shock waves, microjets, turbulence, shear forces, and localized extreme temperatures, which disrupted the integrity of the cell membrane, leading to protein and DNA leakage. US combined with chlorine disinfection could decrease the generation of VBNC bacteria, during which the most disrupted metabolic pathways involved amino acid, carbohydrate, and lipid metabolism. In this figure, red arrows represent upregulation, green arrows represent downregulation, and T-shaped arrows mean suppression effect.

Regarding the pasteurization mechanisms of HPP treatments, it has been reported that several processes, including the disruption of cell structures, the inhibition of key metabolic enzymes, protein denaturation, and interference with genetic material, are involved (Figure 1) [48]. First, many studies have reported that the high pressure of HPP acts mainly on the cell structure. Under scanning electron microscopy (SEM), it was observed that HPP treatments disrupt cell morphology, causing the rupture of the cell membrane, which led to the increase in cell permeability and the release of intracellular substances [49,50,51,52,53]. Additionally, HPP induces phase transitions in the lipid bilayer, a decrease in membrane protein, and protein conformational changes [54]. Secondly, intracellular ribosomes are also one of the key targets in HPP. The increase in pressure disrupts the non-covalent bonds between ribosomal subunits, leading to the dissociation of critical proteins and irreversible ribosomal damage (Figure 1) [55,56]. Lastly, since HPP does not act on covalent bonds, the primary structure of proteins remains un-altered. However, the increased and prolonged HPP treatment affects the higher-order structure of protein, which destabilizes intracellular proteins and results in aggregate formation [25,57,58].

When HPP is combined with other pasteurization methods, synergistic effects are developed. For example, when combining HPP with nisin, HPP-induced damage to the outer membrane could facilitate nisin’s access to its cytoplasmic membrane, thereby enhancing antimicrobial efficacy through forming ion-permeable pores in the cytoplasmic membranes of cells [42]; when combined with a freezing treatment, the lipid bilayer of the microorganisms transformed to the gel phase, and the rigidity of this gel structure was enhanced by the pressure, which resulted in an increased bacterial susceptibility to pressure [37].

### 2.3. VBNC Induction via HPP and Its Mechanisms

The VBNC state refers to a condition in which microbial cells cannot form colonies on routine culture media but maintain metabolic activity and potential virulence. Under suitable conditions, VBNC cells can regain their proliferation ability, causing hazardous risks to food safety [59]. In fact, the VBNC state is always induced under sublethal stress conditions, as lethal exposures result in direct microbial inactivation rather than VBNC formation. In most instances, HPP can completely inactivate microorganisms in foods. Nevertheless, VBNC cells might pose alternative risks. Berlin et al. [60] demonstrated that HPP (200–300 MPa, 25 °C, 5–15 min) effectively inactivated all tested strains of pathogenic *Vibrio* without inducing a VBNC state. However, when the treatment conditions do not reach the threshold for complete microbial inactivation, the microorganisms might enter the VBNC state, which can be inferred through comparisons between plate count-based detection methods and viability-based methods. Ritz et al. [61] reported that HPP (400–600 MPa, 2 °C, 10 min) achieved an 8-log reduction in culturable counts of *L. monocytogenes* and *Salmonella* Typhimurium. However, direct viable counting through the use of epifluorescence microscopy to enumerate elongated, dye-stained cells following antibiotic-induced division inhibition revealed about 4-log surviving cells. Notably, these cells could be resuscitated during subsequent storage at 4 °C and 20 °C [61]. Karamova et al. [62] observed that HPP (300 MPa, 23 °C, 15 min) induced a more than 7-log reduction in culturable counts of *S.* Typhimurium. In contrast, flow cytometry (FCM) analysis through measuring propidium iodide (PI)-excluding cells revealed only a 0.72-log reduction, indicating a substantial population of membrane-intact, potentially viable cells [62]. Yang et al. [63] discovered that VBNC bacteria were present during the whole HPP treatment, and a positive correlation was observed between bacterial pressure resistance and the formation of resuscitable VBNC (RVBNC) populations. Furthermore, combining HPP with CO_2_ or nisin significantly reduced RVBNC cell formation while enhancing bactericidal efficacy [63]. In a recent study, VBNC *Lactiplantibacillus plantarum* was induced through HPP (400 and 500 MPa, 600 s), and VBNC cells began to resuscitate on day 6, with this state delaying post-acidification by at least 24 days. Metabolic analysis revealed different metabolites that were significantly enriched in riboflavin metabolism, in which a significant reduction in flavin mononucleotide and flavin adenine dinucleotide was observed [64]. Coupled with NADH reduction, a key coenzyme in lactic acid production, these effects promoted the induction of a VBNC state in *L. plantarum* [64]. These findings highlight the potential application value of VBNC cells in fermentation control.

## 3. Microbial Inactivation via Pulsed Electric Field (PEF) Technology and Potential Hazardous Risks

### 3.1. Microbial Inactivation via PEF

Pulsed electric field (PEF) pasteurization technology is a technique that utilizes high-intensity (typically 20 to 80 kV/cm) pulsed electric fields to sterilize food products placed between two electrodes within a short duration (μs to ms) [65]. Thanks to its advantages, including its low energy consumption, short processing time, minimal changes in the physicochemical properties of the treated food, and insignificant changes in nutrition and flavor, it is widely favored in the processing of liquid foods containing heat-sensitive substances, such as fruit juices, milk, and liquid eggs. In recent years, technological development has enabled the application of a relatively new type of pulsed electric field—a nanosecond pulsed electric field—in the processing of liquid foods. It requires a much shorter processing time (tens of nanoseconds) and a higher electric field strength (10 million volts per meter), which enable better control over the extreme increase in temperature during the treatment process [66]. The pasteurization effect of PEF technology is usually influenced by various factors, including the electric field intensity, pulse shape, frequency, number of pulses, and duration between pulses, as well as the characteristics of the cells, such as their size and shape and the electrical conductivity of the cytoplasm [67,68]. In addition to these factors, combining PEF with other non-thermal preservation technologies, such as ultrasound, high-pressure processing, and ultraviolet light treatment, can also enhance pasteurization efficiency (Table 3) [42,69,70]. PEF processing is currently used in commercial applications in the United States, Europe, and so on. However, its use in continuous industrial-scale processing is limited by the uniformity of the electric field, and scaling up to industrial levels could result in reduced treatment efficiency [71].

### 3.2. Bactericidal Mechanisms of PEF Pasteurization

The core mechanism of PEF pasteurization is the irreversible electroporation of the cell membrane, commonly referred to as electro-permeabilization. This process involves the application of high-voltage, short-duration electric pulses to induce the formation of pores in the phospholipid bilayer of microbial cell membranes [93,94]. Electroporation primarily consists of three stages. (1) The establishment of transmembrane potential: cells inherently possess a resting transmembrane voltage (TMV); when exposed to an external electric field, the charged molecules within the microbial cell membrane begin to move and accumulate on the membrane surface, creating a transmembrane potential. As the external electric field increases, the cell membrane gradually thins [95,96]. (2) Pore formation: with continuous treatment, structural changes occur in the phospholipid bilayer, leading to the formation of irreversible hydrophilic pores that disrupt the membrane’s barrier function [97,98]. (3) Pore evolution: under the influence of the electric field, the number and size of pores change over time. The formation and evolution of pores are the most critical steps in the PEF pasteurization process [94]. Nevertheless, even low field strength can compromise cell membrane permeabilization. For instance, Loghavi et al. [99] demonstrated that in a moderate electric field fermentation process, *Lactobacillus acidophilus* exhibited significant membrane permeabilization at field strengths as low as 2 V/cm. PEFs can also induce changes in the higher-order structures of protein molecules, which in turn affect the structure and function of membrane proteins [65,94,100,101]. For example, the function of transmembrane ion channels (such as K^+^ and Na^+^ channels) might be disrupted, leading to an imbalance in the concentration of ions inside and outside of the cell. In addition, a PEF might also damage the cytoskeleton, further affecting the integrity and function of the cell [67]. These changes ultimately lead to irreversible damage to the cell membrane, causing cellular contents (enzymes, nucleic acids, proteins, etc.) to leak outside the cell, which disrupts the balance of ion concentrations inside and outside the cell membrane, affecting cellular metabolic activity and ultimately resulting in cell inactivation (Figure 1).

### 3.3. VBNC State Induced via PEF

Certain microorganisms exhibit a differential capacity to enter the VBNC state following PEF treatment. While Rowan (2004) [102] initially documented VBNC induction in PEF-treated *Bacillus cereus* and *L. monocytogenes*, a subsequent work by Yaqub et al. [103] obtained contrasting results, indicating that PEFs could not trigger VBNC state formation in *E. coli*, *B. cereus,* and *L. monocytogenes.* Entry into the VBNC state was also discovered in *Pseudomonas putida* upon PEF treatment, which could facilitate resuscitation in a rich brain–heart infusion medium [104]. The current lack of mechanistic understanding regarding PEF-triggered VBNC state formation represents a critical research gap. Future studies should examine the factors influencing the induction of a VBNC state via PEF processing and the resuscitation of cells in a PEF-induced VBNC state. The pathogenicity of PEF-induced VBNC cells and the formation and resuscitation mechanisms based on omics analysis also need to be further investigated. These aspects are particularly crucial to understanding the potential food safety implications of this physiological state and developing enhanced microbial control strategies in PEF processing.

## 4. Microbial Inactivation of Cold Atmospheric Gas Plasma (CAP) Technology and Hazardous Risks

### 4.1. Microbial Inactivation via CAP

CAP, an emerging non-thermal technology, represents the fourth fundamental state of matter—a dynamic mixture of partially or fully ionized gas [105]. Until now, nearly all studies on microbial inactivation using CAP have relied on inert gases, particularly argon and helium [106]. The most widely used plasma-generation methods are dielectric barrier discharge (DBD) and atmospheric-pressure plasma jets (APPJs) [107]. However, CAP equipment is usually expensive, and due to its small scanning area, its pasteurization effect is highly limited when working with thick, large, and rough materials, resulting in remaining microbial contamination of the product surface [108]. Moreover, the lack of standardized protocols complicates industrial scalability. To overcome this limitation, plasma-activated water (PAW), which is produced through plasma–liquid interactions, has been developed as an effective alternative due to its ability to uniformly treat large-scale food surfaces [108].

Studies have shown that both CAP and PAW are highly effective at eliminating a wide range of microorganisms including bacteria, fungi, and even spores and biofilms in foods (Table 4) [109,110,111,112]. To enhance the inactivation efficiency, oxygen or other reactive gases are often introduced [113]. When external energy sources such as thermal energy, electric fields, magnetic fields, radio frequencies, or microwave frequencies are applied to gases, the electrokinetic energy of the gas atoms increases significantly. This heightened energy initiates a succession of collisions within the gas, ultimately resulting in plasma formation [114].

### 4.2. Bactericidal Mechanisms of CAP

The microbial inactivation efficiency of CAP depends on various factors, including the surface characteristics of different foods, the type of plasma device used, and the type of bacteria [159]. For example, foods with uneven surfaces may require more time for complete bacterial inactivation compared to smoother surfaces [160]. The sensitivity of vegetative forms of planktonic bacteria was notably greater than that of spores or biofilm forms, which required much longer exposure times for inactivation [159]. The antimicrobial efficacy of CAP is primarily mediated by reactive oxygen species (ROS), including superoxide (O^2-^), hydrogen peroxide (H_2_O_2_), hydroxyl radical (·OH), and ozone (O_3_), and reactive nitrogen species (RNS), including nitric oxide (NO), nitrate (NO_3_^−^), nitrite (NO_2_^−^), and peroxynitrite (ONOO^−^), which are produced during CAP treatment [161,162,163]. These generated ROS and RNS could interact with microbial cells to produce a pasteurization effect. For example, in cellular proteins, ROS or RNS mediate microbial inactivation through multiple protein-damaging mechanisms: (1) chemical modifications of amino acid side chains (e.g., excessive disulfide bond oxidation) that impair enzymatic activity [162]; (2) the destruction of cofactors or prosthetic groups, leading to the irreversible inactivation of cofactor-dependent proteins [164]; and (3) the ·OH-mediated cleavage of polypeptide backbones, which disrupts secondary and tertiary structures and permanently compromises protein function [165]. This cumulative oxidative damage ultimately leads to microbial cell death. Beyond protein damage, ·OH was also reported to induce the peroxidation of unsaturated fatty acids in cellular membrane bilayers; this oxidative damage disrupts membrane integrity, resulting in the leakage of intracellular components and ultimately causing microbial cell death [166]. Furthermore, the accumulation of ROS generated during CAP treatment has been shown to induce programmed cell death in bacteria through an alternative sublethal pathway, exhibiting apoptotic-like characteristics, which revealed that microbial inactivation involves not only physical and chemical damage but is also regulated by biological signals [167].

The pasteurization mechanism of CAP treatment varied significantly in dry and aqueous samples (Figure 1). In aqueous environments, which were termed PAW, cytotoxins such as nitric oxide (NO), nitrite (NO_2_^−^), nitrate (NO_3_^−^), and hydrogen peroxide (H_2_O_2_) were generated through plasma–liquid interactions, playing a dominant role in microbial inactivation [125,168]. The generated HNO_2_, HNO_3_, and H_3_O^+^ also caused the solution to acidify. When the pH drops below the critical threshold of 4.7 for microbial inactivation, the combined action of acidic conditions and reactive species synergistically enhances the pasteurization efficacy [169,170].

Under dry conditions, SEM analysis of CAP-exposed cells revealed that cell morphology was altered significantly, including an increase in surface roughness, membrane deformation, and a loss of cellular integrity [171]. This might result from electrostatic forces generated by charged particle accumulation on the outer membrane surface. When these forces exceed the critical membrane rupture threshold, they could induce the structural failure of the membrane, leading to intracellular component leakage and consequent cell inactivation [117,172]. Additionally, UV radiation was also generated during CAP treatment that exhibited a power density below 50 W/cm^2^, suggesting negligible direct bactericidal effects [166]. However, Schneider et al. [173] suggested that the produced UV could interact with the effluent of the He/O_2_ plasma, resulting in accelerated microbial death. UV radiation-induced DNA damage might represent an auxiliary pasteurization mechanism [174].

### 4.3. VBNC State Induced by CAP and Formation Mechanisms

When exposed to CAP treatment, a proportion of *Chromobacterium violaceum* within biofilms was hypothesized to enter a VBNC or dormant state [175,176]. Dolezalova et al. [177] also observed significantly lower *E. coli* counts following plasma treatment (argon, 1–5 kV, 1.5 MHz, 45 min) when assessed via conventional cultivation compared to fluorescence-based staining, suggesting that the bacteria might have entered the VBNC state [177,178]. However, the VBNC cells could not achieve resuscitation [177]. Similarly, Xu et al. [179] also reported that plasma exposure (N_2_, 10 kV, 20 min) brought *Staphylococcus aureus* and *E. coli* into the VBNC state, and more *S*. *aureus* entered this state than *E. coli*, illustrating that the Gram-positive *Staphylococcus aureus* was more resistant to plasma-induced environmental stresses than Gram-negative *E. coli*. *L. monocytogenes* attached to solid surfaces could also enter the VBNC state immediately after CAP treatment, and VBNC cells had the potential to resuscitate and become culturable again, posing risks to public health [180]. Beyond the direct inactivation method of CAP, PAW disinfection can also induce a VBNC state in bacteria. Sun et al. [181] found that PAW treatment induced a VBNC state in five sequence types of *Salmonella* Newport.

While in a CAP-induced VBNC state, cells exhibit enhanced tolerance to external stresses. Liao et al. [182] found that resistance to oxidative and antibiotic stresses in CAP-induced VBNC *S. aureus* was increased. During this process, the expression levels of antioxidative response-related genes including *dps, trxA*, *katA,* and drug efflux pump-related genes of *lmrS* in VBNC cells were significantly increased, revealing that cellular energy depletion, antioxidant responses, and the upregulation of multidrug efflux pump were the major mechanisms (Figure 1) [182]. Regarding pathogenicity, CAP-induced VBNC *S. aureus* maintained its infectious capacity towards HeLa cells, with the upregulated expression of multiple virulence factors (ClfB, SCIN, SdrD, and SasH). This enhanced virulence profile enabled VBNC *S. aureus* to effectively adhere to and internalize within host cells while evading host immune defenses [182]. Although VBNC *S. aureus* loses its replicative capacity, this subpopulation may retain pathogenicity and is frequently underestimated by conventional detection methods. When food products contaminated with VBNC bacteria enter the market, they may pose a significant risk of foodborne disease outbreaks. Therefore, it is important to update food safety guidelines to incorporate the VBNC state into microbial risk assessment frameworks.

Regarding the mechanisms of VBNC induction by PAW, it was revealed that the secretion of outer membrane vesicles was observed in VBNC *S*. Newport, which contributed to the removal of harmful substances. Additionally, the expression of oxidative stress-related genes of *sodA* and *katE*, outer membrane proteins of *ompA/C/F*, and virulence factors of *pagC, sipC* and *sopE2* were significantly upregulated in response to PAW exposure, which imposed a substantial metabolic burden on *S*. Newport, resulting in the severe depletion of intracellular ATP levels [181]. This energy crisis might serve as a key driver for VBNC state formation in *S*. Newport (Figure 1). From a fundamentally different perspective, Borkar et al. [183] demonstrated that plasma-generated nitric oxide water (PG-NOW) prevented the entry of *Micrococcus luteus* into the VBNC state. Mechanism exploration revealed that PG-NOW enhanced the expression of specific homeostasis genes related to growth and metabolism, such as *rpf* (resuscitation promotion factor), *eno* (a fatty acid beta-oxidation-related gene), and *asd* (which is essential for the biosynthesis of aspartate and lysine) [183]. This cellular response may help maintain culturability while inhibiting the transition to the VBNC state [184]. Furthermore, PG-NOW-treated *M*. *luteus* exhibited significantly attenuated pathogenicity in infected human lung cells [183]. Therefore, the PG-NOW treatment could shift VBNC cells into a culturable state susceptible to various treatment strategies, which is essential for managing this clinically challenging bacterial state.

## 5. Microbial Inactivation via High-Pressure Carbon Dioxide (HPCD) Technology and Hazardous Risks

### 5.1. Microbial Inactivation via HPCD

HPCD processing is an innovative non-thermal technology for food preservation. During HPCD treatment, foods are subjected to pressurized CO_2_ (typically 5–50 MPa) at moderate temperatures (<50 °C) [185]. This method effectively inactivates microorganisms and inhibits enzyme activity through synergistic mechanisms, including high pressure, acidification, anaerobic conditions, and explosive decompression, thereby extending shelf life while maintaining food quality [186]. A key advantage of this method is that CO_2_ is non-toxic, non-flammable, cost-effective, and readily available [185]. To date, HPCD technology has been predominantly applied to liquid food matrices, particularly fruit juices and beverages [187]. This is because CO_2_ solubilization requires liquid matrices. However, it may also lead to residual acidity in products due to carbonic acid formation. The antimicrobial efficacy of HPCD treatment depends not only on the microbial species but also on the substrate in which the microorganisms reside (Table 5). Under conditions below 50 MPa and 60 °C, HPCD treatment can achieve a 2–12 log CFU reduction (Table 5). Furthermore, when the treatment temperature is increased to a certain threshold, even bacterial spores can be effectively inactivated (Table 5) [188].

### 5.2. Bactericidal Mechanisms of HPCD

Research on the antimicrobial mechanisms of HPCD has primarily been conducted at the cellular level. Numerous studies have demonstrated that HPCD induces irreversible damage to bacterial cell envelopes. For example, Hong et al. [210] observed that *L. plantarum* treated with HPCD exhibited irreversible membrane damage, manifested as reduced salt tolerance, the leakage of UV-absorbing substances and cellular ions, and an increased uptake of phloxine B dye. In subsequent work, Hong et al. [214] also documented expanded periplasmic space between the cell wall and membrane, membrane rupture, decreased cytoplasmic density, and notably lowered density in cytoplasmic central regions. Liao et al. [215] also reported outer membrane destruction and an increase in cytoplasmic membrane permeability, which was accompanied by reduced membrane fluidity and plasmolysis. Garcia-Gonzalez et al. [216] proposed that HPCD creates pores in cell walls to alter permeability while concurrently damaging intracellular nucleic acids. Additionally, some researchers attributed metabolic suppression in *L. plantarum* to HPCD-induced enzyme inactivation, which resulted from altered membrane permeability and enzyme leakage [217]. Alternatively, it has been suggested that CO_2_ penetration lowers cytoplasmic pH, therefore decreasing the cytoplasmic enzyme activity [185,218,219]. Based on these findings, Garcia-Gonzalez et al. [185] proposed a seven-step mechanism for HPCD antimicrobial action: (1) the dissolution of pressurized CO_2_ in extracellular fluids; (2) membrane modification; (3) cytoplasmic pH decline; (4) cytoplasmic enzyme inactivation; (5) direct metabolic inhibition; (6) electrolyte imbalance; and (7) cell structural degradation.

### 5.3. VBNC State Induced via HPCD and Formation Mechanisms

The induction of a VBNC state in cells via HPCD and their resuscitation have been studied. Initially, Liao et al. [212] observed that the initial culturable count of yeasts and molds in apple juice treated with HPCD (20 MPa, 42 °C, 30 min) fell below the detection limit. However, after 14 days of storage at 2 °C, the microbial counts increased, indicating that the yeasts and molds may have entered the VBNC state following HPCD treatment and were subsequently resuscitated [212]. Later, Zhao et al. [205] verified that *E. coli* O157:H7 were induced into this state by HPCD treatment (5 MPa, 25 °C/31 °C/34 °C/37 °C). During HPCD treatment, elevated temperatures can enhance CO_2_ diffusivity and increase cell membrane fluidity [185]. These effects promote more extensive interaction between CO_2_ and cells, potentially accelerating the loss of culturability. In this state, the *E. coli* O157:H7 cells barely changed size, but their morphology transitioned to a curved rod shape with a relatively rough surface [220]. VBNC cells exhibited enhanced resistance to sonication, which is potentially attributable to modifications in cell-wall composition, including increased peptidoglycan cross-linking [221].

The mechanisms of VBNC state formation were further investigated. Global metabolic analysis revealed that genes and proteins associated with membrane transport, central metabolism, DNA replication, and cell division were downregulated in *E. coli* O157:H7 VBNC cells. Additionally, pathogenicity-related genes and proteins exhibited reduced expression, aligning with the observed decline in the capability of VBNC bacteria to adhere to HeLa cells [222]. Among the multiple synergistic effects of HPCD, high pressure was found to accelerate VBNC formation, whereas acidification (pH 3) was the main factor for the induction of a VBNC state in *E. coli* O157:H7. This conclusion was supported by the observation that cells in an acid-induced and HPCD-induced VBNC state exhibited similar stress resistance characteristics, including 59 differentially expressed genes involved in cellular transport and localization [223]. Consistently, Pan et al. [209] found that *asr*, encoding an acid shock protein, was the most induced gene following HPCD treatment (5 MPa, 25 °C, 30 min). The high expression of *asr* suppressed acid resistance systems, leading to intracellular proton accumulation. It concurrently downregulated the expression of *hchA*, a key protein stabilization factor. These dual effects collectively promoted endogenous protein aggregation, which showed a positive correlation with VBNC state formation (Figure 1) [224]. Further investigation demonstrated that intracellular ATP levels played a crucial role, showing a strong negative correlation with VBNC formation. Moreover, preadaptation to heat, acidic conditions, and extended cultivation (24 h) were found to facilitate VBNC induction by significantly reducing intracellular ATP levels [225]. The cell division-related genes of *dicC* and *dicA* also contributed to entry into a VBNC state. The gene of *dicC* acts as a negative regulator of VBNC state formation, while *dicA* promotes cellular entry into the VBNC state. Furthermore, the regulation of cell growth rate by *dicC* and *dicA* coupled with morphological dwarfing was positively associated with VBNC state formation (Figure 1) [226]. The outer membrane protein OmpF was also involved in the formation of the VBNC state, since the overexpression of OmpF promoted VBNC state entry [224]. However, the precise molecular mechanisms underlying specific regulatory effects require further investigation.

The resuscitation of cells in an HPCD-induced VBNC state was also confirmed. Zhao et al. [220] revealed that HPCD-induced VBNC *E. coli* (5 MPa) achieved resuscitation at 25 °C, 31 °C, and 34 °C with incubation in tryptic soy broth at 37 °C, indicating significant hazardous risks. Under more severe induction conditions (5 MPa, 37 °C), VBNC cells underwent irreversible cell death rather than resuscitation, which might be due to the more extensive cellular damage caused by a harsh environment. Regarding the resuscitation mechanisms of HPCD-induced VBNC cells, Yang et al. [227] found that the mutation of *rfaL*, which encodes an O-antigen ligase, markedly shortened the resuscitating lag phase to promote the resuscitation of VBNC *E. coli*. A further mechanism study revealed that Δ*rfaL* VBNC cells contained higher levels of ATP, which was used to activate the Handler and salvage pathways to synthesize NAD^+^ to recover cell activity. A resuscitation strategy was finally proposed, stating that VBNC cells utilize residual ATP to restore metabolic activity, thereby exiting dormancy. The synthesis pathway of lipopolysaccharide in a *rfaL* null mutant was inhibited and could supply more ATP to support NAD+ synthesis and consequently promote resuscitation [227]. It is biologically plausible that the activation of the resuscitation process requires ATP participation; therefore, a resuscitation mechanism for VBNC *Escherichia coli* mediated by *rfaL* depletion and ATP supplementation may represent a universal pathway across various non-thermal technologies.

## 6. Microbial Inactivation via Ultrasound (US) Technology and Hazardous Risks

### 6.1. Microbial Inactivation via US

US refers to acoustic waves with frequencies above 20 kHz, which is beyond the upper limit of human hearing [228]. According to intensity and frequency, US can be classified as low intensity (LIU; 100 kHz–1 MHz, <1 W/cm^2^) and high intensity (HIU; 20–100 kHz, >1 W/cm^2^) [229]. While LIU has predominantly been employed to detect and secure the quality of food [230], to date, US-assisted microbial inactivation has garnered increasing research interest due to its operational simplicity, low energy, and water requirements, and remarkable capacity to preserve the nutritional quality and sensory attributes of food products [231].

The antimicrobial efficacy of US is influenced by various factors, including treatment duration, bacterial strain characteristics, and the composition and properties of the food matrix [232]. For example, Gram-positive bacteria exhibit a higher resistance to ultrasound compared to Gram-negative bacteria due to their thick cell walls and tight adhesion to the peptidoglycan layer [233,234]; rod-shaped bacteria are more sensitive than coccoid forms [235]; aerobic bacteria display greater resistance than anaerobic species [235]; and bacterial spore formers demonstrate a higher tolerance than vegetative cells [236]. Regarding the influence of food composition, it is noteworthy that certain food matrices exhibit a protective effect against US treatment. For instance, whole milk demonstrated a greater reduction in the bacterial inactivation efficacy of US compared to fat-free milk (Table 6) [237]. Thus, US is always combined with antimicrobial substances or other approaches like high pressure, UV light, pulsed electric fields, and heating at mild temperatures to increase its antimicrobial efficiency (Table 6). However, the practical application of ultrasound (US) technology is limited by several factors, including high equipment costs, non-uniform treatment effects to some extent, and the generation of free radicals due to localized extreme temperatures during cavitation.

### 6.2. Bactericidal Mechanisms of US

The cavitation effect is central to US processing and involves bubble formation, growth, and collapse [257,258]. Ultrasonic waves induce sudden pressure drops in the liquid medium, nucleating microscopic bubbles. As the pressure fluctuates, these bubbles expand until they reach a critical size and then implode violently. This collapse generates shock waves, microjets, turbulence, shear forces, and localized extreme temperatures/pressures [259]. The intense heat decomposes water to generate reactive species and hydrogen peroxide, which react with the DNA of microorganisms and disrupt the replication process [260]. Simultaneously, intracellular oxidative stress is increased, which triggers bacterial stress responses and causes metabolism suppression [260,261]. In a study of the morphology and metabolism of *E. coli* O157:H7, Lin et al. [261] also demonstrated that US treatment disrupted cell membrane integrity, leading to protein and DNA leakage and a decrease in metabolism-related enzyme activities. Beyond its direct bactericidal effects, researchers discovered that US can create transient pores in bacterial membranes, facilitating enhanced penetration of antimicrobial agents into cells. These temporary channels close immediately upon cessation of US [262]. Therefore, these synergistic mechanisms collectively contribute to US’s antimicrobial efficacy.

Furthermore, US treatment also demonstrated significant biofilm-disrupting capabilities. The formation of micropores in biofilms induced by US enhanced bacterial susceptibility to antimicrobial agents, thereby improving bactericidal efficacy [263]. Additionally, US promoted the transport of oxygen and nutrients within biofilms and accelerated the removal of bacterial metabolic byproducts. These effects might reactivate dormant bacteria in biofilms and ultimately accelerate biofilm disintegration [264].

### 6.3. VBNC State Induced via US and Formation Mechanisms

The published research addressing the effects of ultrasound exposure on VBNC state induction is remarkably sparse. Declerck et al. [265] demonstrated that US treatment (36 kHz, 50% power setting) induced approximately 7% of *Legionella pneumophila* populations to enter the VBNC state, rendering them undetectable by conventional culture methods. Moreover, regarding pathogenicity, VBNC *L. pneumophila* also remained virulent [266], posing significant hazardous risk [265]. US treatment was also combined with other methods to increase its microbial inactivation efficiency. Li et al. [267] found that a single instance of US treatment (20 kHz, 13.3 W/mL, 20 °C, 10 min) could induce 45.75% *S. aureus* into the VBNC state, while US combined with slightly acidic electrolyzed water reduced the proportion to 0.07%, showing synergistic effects. This was due to greater damage to the ultrastructure of *S. aureus* [267]. Similarly, the number of VBNC *Pseudomonas aeruginosa* induced by US and chlorine treatment was 10^3.6^ CFU/mL and 10^5.2^ CFU/mL, respectively, while US combined with chlorine disinfection generated 10^1.3^ CFU/mL VBNC bacteria, and the reactivation of the VBNC cells was effectively suppressed. Metabolomic analysis revealed that US/chlorine treatment significantly affected the glutathione and cysteine metabolism of *P. aeruginosa*, which decreased the production of superoxide dismutase, preventing the bacteria from maintaining a normal redox balance [268]. Moreover, the significant decrease in pyruvate metabolism indicated that the synthesis pathway of pyruvate, the major antioxidant, was significantly disturbed, leading to the accumulation of ROS and increased lipid peroxidation [268]. Finally, the excessive oxidative stress led to bacterial death rather than VBNC induction.

To date, no studies have explored the underlying mechanisms of a US-induced VBNC state. We propose that bacterial cells may face three potential outcomes following this treatment: (1) lethal damage, resulting in immediate cell death; (2) sublethal injury, caused by physical inactivation effects, ultimately leading to delayed death; (3) entry into the VBNC state, which we hypothesize is most likely associated with the generation of reactive species and hydrogen peroxide (as discussed in Section 6.2). In this scenario, elevated intracellular oxidative stress triggers bacterial stress responses, ultimately suppressing metabolic activity and inducing a VBNC state. The specific mechanisms need to be further investigated.

## 7. Regulatory and Detection Challenges Associated with VBNC Cells

Considering the potential risks of VBNC cells for food safety and public health, the development of reliable detection methods is of great importance. When bacteria enter the VBNC state, due to their nonculturable nature, traditional microbiological detection techniques (e.g., plate counting) fail to identify them, leading to the potential underestimation of microbial risk. Consequently, alternative detection strategies have been developed, which primarily target markers of viability rather than culturability. VBNC cell detection methods have been widely reviewed, among which PMA-qPCR and methods based on fluorescence staining combined with flow cytometry are the most widely adopted in VBNC studies [13,269]. These two methods were developed according to the membrane-intact characteristics of VBNC cells. The PMA dye can pass through the compromised membrane to bind with DNA and inhibit its amplification; therefore, viable or VBNC cells can subsequently be quantified via qPCR [13]. Additionally, the LIVE/DEAD *Bac*light assay based on fluorescence staining utilizes a dual-staining approach with SYTO 9 (a green fluorescent dye) and propidium iodide (PI, red fluorescent). SYTO 9 permeates both intact and damaged membranes, whereas PI exclusively enters cells with compromised membranes, where it competitively displaces SYTO 9 by binding to nucleic acids [220]. The stained cells can then be analyzed via flow cytometry. However, in an actual detection environment, flow cytometry equipment struggles to satisfy the detection requirements due to the complicated composition of real food samples. Additionally, the above methods cannot distinguish between viable cells and VBNC cells since they all possess an intact cell membrane. Therefore, to accurately assess microbial risk in practical applications, VBNC cells should be incorporated into viable microorganisms using molecular detection techniques such as PMA-qPCR, which can prevent the underestimation of potentially hazardous microbial populations in foods.

## 8. Integrative Comparison of Cells in a VBNC State Induced by Different Non-Thermal Technologies

The pasteurization effects of different non-thermal processing technologies are summarized and compared in Table 7. Generally, HPP and PEF processing cause minimal changes to the physicochemical properties of food products. On the other hand, during CAP and US treatments, free radicals are generated, which might cause oxidation in food and therefore affect its qualities. Due to their treatment advantages, HPP and PEFs have commercial applications (Table 7). Regarding VBNC formation potential, the four technologies all reported the generation of VBNC bacteria; however, the research results were inconsistent for PEF-induced VBNC induction, which requires further investigation. CAP/HPCD/US-induced VBNC cells may remain virulent (Table 7). Therefore, treatment parameters should be optimized to higher intensities to ensure the complete inactivation of VBNC bacteria.

While different non-thermal technologies impose distinct stress conditions on microorganisms, leading to diverse bacterial responses and treatment-specific formation mechanisms, the resulting VBNC states exhibit certain common characteristics in their formation pathways, among which ATP depletion is the most important. For example, the riboflavin metabolism of HPP-treated *L. plantarum* was disrupted, impairing the cellular respiratory chain and ATP generation [64]; during CAP treatment, more than half of the metabolic enzymes (e.g., arylamidase, glucosidase, and galactosidase) in *S. aureus* were suppressed, indicating remarkable metabolism suppression and ATP depletion [182]; prior to the treatment of HPCD, although ATP concentration was decreased through the exposure of *E. coli* to carbonyl cyanide m-chlorophenyl hydrazine (CCCP), the number of HPCD-induced VBNC cells increased [225]. Therefore, maintaining low cellular energy levels is a survival strategy that enables VBNC cells to preserve their viability. Apart from these, some mechanisms are treatment-specific. For example, because CAP is able to generate reactive species, the oxidative stress responses of bacteria, such as the increased expression of antioxidant enzymes, are triggered [181,182], which contributes to the survival of bacteria through VBNC state entry. Furthermore, given that acidification constitutes a key mechanism in HPCD pasteurization, our previous research demonstrated that HPCD treatment activates *asr* gene expression, which subsequently initiates multiple pathways leading to cellular protein aggregation and ultimately promoting VBNC state entry [224]. Collectively, future investigations into VBNC state induction mechanisms should incorporate the specific effects of different pasteurization methods, and the shared mechanisms of VBNC induction can also inform subsequent inferences.

## 9. Conclusions and Future Perspectives

As innovative microbial inactivation approaches, non-thermal processing technologies have garnered significant attention due to their profound antimicrobial effects. However, their potential to induce a VBNC state poses hidden risks that cannot be overlooked. Although the pathogenicity of VBNC cells remains inconclusive, accumulating evidence suggests that certain VBNC pathogens retain virulence factors and maintain the ability to invade host cells, posing a potential food safety threat. This review systematically summarizes the microbial inactivation efficiency and mechanisms of common non-thermal technologies alongside their potential to induce a VBNC state. By elucidating the risks associated with these advanced technologies despite their high inactivation efficiency, this review aims to provide insights for further optimization and advancement in non-thermal food processing technologies.

Notably, research progress on VBNC induction by various non-thermal processing technologies has been highly inconsistent. Most studies have been devoted to discovering cell entry into the VBNC state, and mechanism exploration has remained limited. Further studies can strengthen such research through omics-based analysis and verification; an investigation of the functions of specific genes/proteins; and the coupling of resuscitation and induction mechanisms. Regarding the detection methods, more bio-markers can be developed to distinguish VBNC cells and resuscitating VBNC cells from other cell populations. Moreover, current research in this field remains predominantly theoretical, warranting further translation into practical applications for better controlling the risks posed by VBNC bacteria—for example, establishing a predictive model of VBNC state induction and resuscitation in bacteria or assessing the hazard threshold levels of VBNC pathogens.

## Figures and Tables

**Table 2:** Microbial inactivation effects of HPP in different foods.

Treatment	Food Sample	Microorganism	Inactivation (log*N*_0_/*N*)	Condition	Reference
HPP	Green onions and salsa	Human Norovirus	>3	600 MPa/1 °C/2 min	[26]
	Oysters and clams	Human Norovirus	>4	450 MPa/1 °C/5 min	[27]
	Strawberries	Murine norovirus (MNV-1)	5.8	450 MPa/pH 4.0/4 °C	[28]
	Strawberry puree		4.7		
	Pitaya juice	*Listeria innocua* *Saccharomyces cerevisiae*	>5	550 MPa/16 min 600 MPa/12 min	[29]
	Tryptic soy broth supplemented with 2.5% NaCl	*Vibrio parahaemolyticus*	9	300 MPa/10 min	[30]
	Oysters	*Vibrio parahaemolyticus*	5	≥350 MPa/1 °C–35 °C/2 min ≥300 MPa/40 °C/2 min	[31]
	Cantaloupe puree	*Salmonella enterica* *Listeria monocytogenes*	>6.7	500 MPa/8 °C/5 min	[32]
	Cabbage kimchi	Norovirus	0.1–1.5	100–400 MPa	[33]
	Raw chicken breast filets	*Salmonella* Typhimurium DMST 28913	4	400 MPa/30 °C/1 min	[34]
	Carrot and grapefruit juices	*Escherichia coli* O157:H7	6.4–8.34	615 MPa/15 °C/2 min	[35]
HPP + freezing	Orange juice	*Escherichia coli* K12	4.88	250 MPa/−80 °C/pH 3.2/15 min	[36]
HPP + freezing	Milk	*Escherichia coli* ATCC 25922	3.8	300 MPa/−3 °C/30 s	[37]
HPP + freezing	Frozen beef mince	*Escherichia coli* ATCC 25922	3	300 MPa/5 min	[38]
HPP + liquid smoke + freezing	Raw and hot smoked trout filets	*Listeria monocytogenes*	5	200 MPa/15 min	[39]
HPP + freezing	Frozen chicken breast	*Salmonella*	>3	400 MPa/5 min 500 MPa/1 min	[40]
HPP + food-grade antimicrobials	Raw ground chicken meat	*Salmonella*	5	350 MPa/0.05% AITC/4 min	[41]
		*Salmonella*	>7	350 MPa/0.075% AITC/0.1% AA/12 min	
HPP + nisin + mild thermal	Carrot juice	*Listeria innocua* *Escherichia coli*	7	500 MPa/20 °C/2 min	[42]
HPP + mild heat	Oysters	*Vibrio parahaemolyticus*	>3.52	200 MPa/21 °C/2 min and 45 °C/10 min 200 MPa/21 °C/2 min and 50 °C/2 min 250 MPa/21 °C/2 min and 40 °C/10 min 250 MPa/21 °C/2 min and 45 °C/5 min 300 MPa/21 °C/2 min and 50 °C/5 min	[43]
		*Vibrio vulnificus*	>3.52	200 MPa/21 °C/2 min and 40 °C/10 min 200 MPa/21 °C/2 min and 45 °C/5 min 200 MPa/21 °C/2 min and 50 °C/2 min 250 MPa/21 °C/2 min and 50 °C/10 min	
HPP + CO_2_	Luria–Bertani	*Escherichia coli*	>8	300 MPa/1.2 NL/L CO_2_ 250 MPa/3.2 NL/L CO_2_	[44]
		*Staphylococcus aureus*	>7	350 MPa/3.8 NL/L CO_2_	
HPP + CocoanOX	Liquid Whole Egg and Skim Milk Mixed Beverage	*Bacillus cereus*	3.860	200 MPa/15 min	[45]
HPP + lactoperoxidase system	Beef carpaccio	*Salmonella* Enteritidis	5.4	50 MPa/5 min	[46]
		*Escherichia coli* O157:H7	4.7		
HPP + dense phase carbon dioxide	Feijoa Puree	*Escherichia coli*	4.3	400 MPa/4 min	[47]

**Table 3:** Microbial inactivation effects of PEF in different foods.

Treatment	Food Sample	Microorganism	Inactivation (log*N*_0_/*N*)	Condition	Reference
PEF	Melon juices	*Salmonella* Enteritidis	3.71 ± 0.17	217 Hz/35 kV/cm/4 μs puls/1440 μs	[72]
		*Escherichia coli*	3.7 ± 0.3		
		*Listeria monocytogenes*	3.56 ± 0.26		
	Watermelon juices	*Salmonella* Enteritidis	3.56 ± 0.12	188 Hz/35 kV/cm/4 μs puls/1727 μs	
		*Escherichia coli*	3.6 ± 0.4		
		*Listeria monocytogenes*	3.41 ± 0.13		
	Freshly squeezed orange juice	*Salmonella* Typhimurium	5.9	90 kV/cm/50 μs puls/55 °C	[73]
	Pineapple juice–coconut milk	*Escherichia coli*	5	235–588 Hz/10–21 kV/cm	[74]
		*Listeria innocua*	3.9		
	Raspberry juice	Molds	0.92–2.12	11.3–23.3 kV/cm/10–500 Hz	[75]
		Yeasts	1.38–3.19		
	Orange juice	*Staphylococcus aureus*	5.89–5.924	1 Hz/20–40 kV/cm/100–500 µs	[76]
		*Escherichia coli*	5.876–5.949		
	Milk	Pseudomonads	>5	31 kV/cm/55 °C	[77]
	Tropical Fruit Smoothie	*Escherichia coli*	4.2	34 kV/cm	[78]
	Orange juice	*Pichia fermentans*	4.8	40 kV/cm/100 μs	[79]
		*Listeria innocua*	3.7		
		*Escherichia coli* K12	6.3		
	Citrate–phosphate buffer	Bacterial viable counts	2.8	20 kV/cm/pH 4.0/200 µs	[70]
	Grape juice	*Kloeckera apiculata*	2.24–3.94	35 kV/cm/1 µs	[80]
		*Saccharomyces cerevisiae*			
		*Lactobacillus plantarum*			
		*Lactobacillus hilgardii*			
		*Gluconobacter oxydans*			
	Apple juice	*Escherichia coli* O157:H7	0.4–3.6	20–30 kV/cm/5–125 μs	[81]
	Low-fat Milk	*Escherichia coli*	4.5	200 kJ/L	[82]
		*Saccharomyces cerevisiae*	6.0		
		*Lactobacillus brevis*	4.4		
	Water with salt	*Escherichia coli*	1	107 Vm^−1^/60 × 10^−9^ s	[66]
	Liquid whole egg	*Listeria innocua*	3.5	3.5 Hz/50 kV/cm/32 pulses	[83]
PEF + nisin	Liquid whole egg	*Listeria innocua*	4.1	3.5 Hz/50 kV/cm/32 pulses/10 IU nisin/ml	
			5.5	3.5 Hz/50 kV/cm/32 pulses/100 IU nisin/ml	
	Whey	*Listeria innocua*	4.5	12 kV/cm/50 IU/mL nisin/12 ms	[84]
PEF + heat	Mixed mandarin and Hallabong tangor juice	Aerobe	3.9	16 kV/cm/100 kJ/L/70 °C	[85]
		Yeast/mold	4.3		
		Coliform	0.8		
	Tropical Fruit Smoothie	*Escherichia coli*	6.9	34 kV/cm/55 °C	[78]
			5.1	34 kV/cm/45 °C	
PEF + UV-light	Apple juice	*Escherichia coli* K12	4	20 kHz/15 kV/cm/170 µs/25 °C	[69]
PEF + nisin	Orange juice	*Listeria innocua*	5.6	40 kV/cm/2.5 ppm/100 µs	[79]
		*Escherichia coli* K12	7.9		
PEF + lactic acid		*Listeria innocua*	6.1	40 kV/cm/500 ppm/100 µs	
		*Pichia fermentans*	7.8		
PEF + temperature	Cantaloupe Juice	*Saccharomyces cerevisiae*	>5.0	20 kV/cm/200 µs/55 °C/5 min	[70]
PEF + ethyl lauroyl arginate	Apple juice	*Escherichia coli* O157:H7	0.9–6.7	20–30 kV/50 ppm/5–125 µs	[81]
PEF + US	Spinach juice	*Escherichia coli*/coliform	1.15	9 kV/cm/1 kHz/335 µs + 200 W/40 kHz/30 ± 2 °C	[86]
		Yeast and mold	2.01		
PEF + US	Oil-field re-injection water	Saprophytic bacteria	1.82	2.7 kV/cm/40 kHz/30 min	[87]
		Iron bacteria	2.54	2.7 kV/cm/40 kHz/12 min	
		Sulfate reducing bacteria	1.95	2.7 kV/cm/40 kHz/16 min	
PEF + UV	Apple and cranberry juice	*Escherichia coli* and *Pichia fermentans*	6	34 kV/cm/18 Hz/93 µs + 5.3 J/cm^2^	[88]
PEF + HPCD	McIlvaine buffer solution	*Escherichia coli*	5.74	12 kV/cm/40 J/mL/25 °C + 8.0 MPa/11 min	[89]
PEF + HPP	Water	*Listeria innocua*	>3	30 kV/10^−3^ s + 400 Mpa/100 s	[90]
PEF + TS	Ringer’s solution	*Pseudomonas fluorescens*	48%	29 kV/cm + 18.6 mm	[91]
		*Escherichia coli*	64.8%	32 kV/cm + 18.6 mm	
			71.5%	32 kV/cm + 27.9 mm	
PEF + TS	Beer	*Staphylococcus aureus*	6.8	40 kV/cm/150 µs + 55 °C/10 min	[92]

TS: thermosonication.

**Table 4:** Microbial inactivation effects of CAP in different foods.

Treatment	Food Sample	Microorganism	Inactivation (log*N*_0_/*N*)	Condition	Reference
CAP	Lettuce	*Salmonella enterica* serovar Typhimurium	2.72	12L/min/<35 °C/15 min	[115]
	Strawberry		1.76		
	Potato		0.94		
	Corn salad leaves	*Escherichia coli* O104:H4	3.3 ± 1.1	17 mm/2 min	[116]
	Apple juice	*Citrobacter freundii*	5	Argon and 0.1% oxygen/480 s	[117]
	Tryptic soy agar plates	Methicillin-resistant *Staphylococcus aureus*	4–5	10 min	[118]
	Cress seeds	*Escherichia coli*	3.4	10 kHz/8 kV/500 ns/10 min	[119]
	Almonds	*Escherichia coli* O157:H7 C9490	1.34	6 cm/20 s	[120]
	Non-fat dry milk	*Cronobacter sakazakii*	1.17–3.27	20–120 s	[121]
	Eggshells	*Salmonella enterica*	>5	655 W/120 s	[122]
	Almonds	*Escherichia coli* 12955	5	30 kV/2 kHz/30 s	[123]
	Golden delicious apples	*Salmonella stanley*	2.96–3.72	40L/min/3 min	[124]
		*Escherichia coli* O157:H7	3.4–3.6		
	Tofu	*Salmonella enterica* serovar Typhimurium	0.2–0.6	15 min	[125]
		*Escherichia coli* O157:H7			
	Red pepper powder	*Aspergillus flavus*	2.5 ± 0.3	900 W/667 Pa/20 min	[126]
	Cheese	*Escherichia coli*	4.75 ± 0.02	50 W/10 min	[107]
		*Listeria innocua*	0.72 ± 0.01		
	Blueberries	Total aerobic bacteria	0.34–1.24	12 kV/5 kHz/60 s	[127]
		Mold populations	0.57–0.87		
	Korean rice cakes	*Salmonella*	3.9 ± 0.3	26 kV/3 min	[128]
		Yeast and molds	1.7 ± 0.3		
		Mesophilic aerobic bacteria	2.0 ± 0.2		
	Ready-to-eat ham in modified atmospheric packaging	*Listeria monocytogenes*	4	30 kV/3.5 kHz/10 min	[129]
	Korean steamed rice cakes packaged in plastic pouches	*Escherichia coli* O157:H7	2.2 ± 0.2	30 W/4 min	[130]
		*Bacillus cereus* spores	1.4 ± 0.2		
		*Penicillium chrysogenum*	2.2 ± 0.3		
		Indigenous aerobic bacteria	1.1 ± 0.2		
		Yeast and molds	1.0 ± 0.1		
	Apple juice	*Escherichia coli*	3.98–4.34	30–50 W/<40 s	[131]
	Black peppercorns	*Salmonella*	4.5–5.5	60–80 s	[132]
	Chicken breast	Natural microflora of chicken	2	100 kV/5 min	[133]
	Radish sprouts	*Salmonella*	2.6 ± 0.4	900 W/667 Pa/20 min	[134]
	Lettuce	*Listeria monocytogenes* biofilm	3.85 ± 0.12	750 mJ/cm^2^	[135]
	Cabbage		4.09 ± 0.12		
	Lettuce	*Salmonella* biofilms	4.0 ± 1.3	80 kV_RMS_/300 s	[136]
		*Listeria monocytogenes* biofilms	3.5 ± 0.8		
		*Escherichia coli* biofilms	3.0 ± 2.0		
HVACP	Coconut water	*Salmonella enterica* serovar Typhimurium LT2	1.30	90 kV/120 s	[137]
HVACP	Tilapia filets	Total viable bacteria	7.15	70 kV/5 min	[138]
		*Pseudomonas*	6.99		
		*Enterobacteriaceae*	4.23		
DACP	Romaine lettuce packaged	*Escherichia coli* O157:H7	1	34.8 kV/1.1 kHz/5 min	[139]
DACP	Tomatoes	*Salmonella*	3.3 ± 0.5	35 kV/1.1 A/3 min	[140]
DACP	Bulk romaine lettuce	*Escherichia coli* O157:H7	0.4–0.8	42.6 kV/10 min	[141]
APPJ	Raw chicken breasts	*Escherichia coli*	1.85 ± 0.051	20 mm/10 min	[142]
APPJ	Table eggs	*Salmonella enterica*	7	800 W/20 mm/120 s	[143]
US + CAP	Deionized water	*Escherichia coli* and yeast	6	AC (13 kV/60 Hz) US (140 W/47 kHz)	[144]
Hydrothermal treatment + CAP	Strawberry juice	Total bacterial count	2	60 kV/10 min	[145]
PAW	Kale	*Escherichia coli*	3.48	10 kV/20 kHz/30 min	[146]
PAW	Grapes	*Saccharomyces cerevisiae*	0.38 ± 0.17	8.2 kV/1.1–1.3 mA/30 min	[147]
			0.53 ± 0.07	8.2 kV/1.1–1.3 mA/60 min	
PAW	Button mushrooms, Agaricus bisporus	Bacteria	1.5	10 min	[148]
		Fungi	0.5		
PAW	Celery	*Listeria monocytogenes*	0.57	DBD19.15 V/60 min	[149]
			0.35	DBD19.15 V/30 min	
		*Escherichia coli*	0.57	DBD19.15 V/60 min	
	Radicchio	*Listeria monocytogenes*	2.2	DBD19.15 V/60 min	
			1.8	DBD19.15 V/30 min	
		*Escherichia coli*	1.3	DBD19.15 V/30 min	
PAW	Iceberg lettuce	*Listeria innocua*	2.4	20 kV/5 min	[150]
PAW	Beef	Fungi and yeast	1.76	APPJ 600 W	[151]
PAW	Kumquat	*Penicillium italicum*	0.75	30 min	[152]
			1.3	45 min	
			3.3	60 min	
PAW	Yellow River Carp (Cyprinus carpio) Filets	*Shewanella putrefaciens*	1.03	6 min	[153]
PAW	Ready-to-use shredded salted kimchi cabbage	Mesophilic aerobic bacteria	2.0	120 s	[154]
		Lactic acid bacteria	2.2		
		Yeast and molds	1.8		
Mild heating + PAW		Coliforms	0.9	60 °C/120 s	
		*Listeria monocytogenes*	3.4		
		*Staphylococcus aureus*	3.7		
Mild heating + PAW	Grapes	*Saccharomyces cerevisiae*	5.85	55 °C/30 min	[155]
Thermo-U + plasma + PAW	Grass carp	*Shewanella putrefaciens*	4.40	66 V/60 °C/14.90 min	[156]
		*Salmonella* Typhimurium	3.97		
DBD + PAW	*Lates calcarifer*	Total viable count	1.68	PAW 150 s/DBD 160 kV/180 s	[157]
US + PAW	Chicken meat and skin	*Escherichia coli* K12	1.33	Sample thickness of 4 mm/40 °C/60 min	[158]
		*Staphylococcus aureus*	0.83		

APPJ: atmospheric pressure plasma jet; CAP: cold atmospheric gas plasma; DACP: dielectric barrier discharge atmospheric cold plasma; DBD: dielectric barrier discharge; HVCAP: high voltage atmospheric cold plasma; PAW: plasma-activated water.

**Table 5:** Microbial inactivation effects of HPCD.

Treatment	Sample	Microorganism	Inactivation (log*N*_0_/*N*)	Condition (MPa/℃/min)	Reference
HPCD	Physiological saline	*Saccharomyces cerevisiae*	7.5	20/35/120	[189]
	Growth medium		7	6.9/35/15	[190]
	Sterile water		8	4/40/>180	[191]
	Physiological saline		6	25/35/30	[192]
	Sterile water		8	15/40/60	[193]
	Growth medium		9	6/35/15	[194]
	TSB w/polymers		9	20.5/40/240	[195]
	Orange juice		12	15/25/<10	[196]
HPCD	Physiological saline	*Escherichia coli*	6.5	20/35/120	[189]
	Nutrient broth		2	6.21/RT/120	[197]
	Physiological saline		6	5/35/20	[198]
	Growth medium		9	6/35/15	[194]
	TSB w/polymers		8	20.5/34/30	[195]
	Sterile water		8.7	7.5/24/5.2	[199]
	Orange juice		>6	15/24/4.9	[196]
	Spinach leaves		5	10/40/40	[200]
HPCD	Orange juice	*Escherichia coli* O157:H7	5	10.7/25/10	[196]
	Apple juice		5.7	20.6/25/12	
HPCD	Peptone water	*Escherichia coli* O157:H7	>7	20/45/15	[201]
HPCD	Physiological saline	*Staphylococcus aureus*	5	20/35/120	[192]
	Nutrient broth		2	6.21/RT/120	
	BHIB		7	8/25/60	[202]
HPCD	Distilled water	*Listeria monocytogenes*	9	6.18/35/12	[203]
	Physiological saline with broth		6.98	6/35/75	[204]
	Orange juice		6	38/25/10	[196]
	Peptone water		>7	20/45/15	[201]
HPCD	TSB w/polymers	*Listeria innocua*	9	20.5/34/36	[195]
HPCD	Physiological saline	*Aspergillus niger*	5	20/35/120	[189]
HPCD	TSB w/polymers	*Bacillus cereus*	8	20.5/60/240	[195]
HPCD	Physiological saline	*Bacillus subtilis*	7	7.4/38/2.5	[205]
HPCD	Aqueous solution	*Bacillus subtilis* spores	7	10–15/86/60 6.5–15/91/60	[206]
	Aqueous solution with 0.02% nisin		4.1	20/84–86/30	[207]
HPCD	Pysiological saline	*Brocothirix thermosphacta*	5.5	6.05/35/100	[204]
	Skinned meat		5	6.05/35/150	
HPCD	Physiological saline	*Enterococcus faecalis*	8	6.05/35/18	[204]
HPCD	Growth medium	*Leuconostoc dextranicum*	>8	6.9–20.7/35/15–20	[208]
HPCD	Physiological saline	*Lactobacillus brevis*	6	25/35/30	[192]
HPCD	Growth medium	*Lactobacillus brevis*	9	6/35/15	[194]
HPCD	MRS broth	Lactic acid bacteria	5	6.9/30/200	[209]
HPCD	TSB w/polymers	*Legionella dunnii*	4	20.5/40/90	[195]
HPCD	Growth medium	*Lactobacillus plantarum*	>6	13.8/30/30	[209]
HPCD	MRS broth	*Lactobacillus plantarum*	>8	7/30/100	[210]
HPCD	Orange juice	*Lactobacillus plantarum*	>8	7.5/35/<10	[196]
		*Leuconostoc mesenteroids*	>6	15/25/<10	
HPCD	TSB w/polymers	*Pseudomonas aeruginosa*	8	20.5/40/240	[195]
HPCD	TSB w/polymers	*Proteus vulgaris*	8	20.5/34/36	[195]
HPCD	Nutrient broth	*Salmonella seftenberg*	2	6.21/RT/120	[189]
HPCD	Orange juice	*Salmonella* Thyphimurium	6	38/25/10	[196]
HPCD	Physiological saline		7	6/35/15	[211]
HPCD	Peptone water		>7	20/45/15	[201]
HPCD	TSB w/polymers	*Salmonella* Salford	9	20.5/40/240	[195]
HPCD	Growth medium	*Torulopsis versatilis*	9	6/35/15	[194]
HPCD	Apple juice	Aerobic bacteria	>3.5	20/52/30	[212]
		Yeasts and molds	3.9	20/57/30	
HPCD	Physiological saline	*Escherichia coli*	99.45%	6.5/10/15	[213]
		*Staphylococcus aureus*	94.6%		
	DMEM medium	SARS-CoV-2 spike pseudovirus	>99%		
		Human coronavirus 229E	>1-log virus tilter reduction		

BHIB: Brain-Heart Infusion Broth; MRS: De Man Rogosa Sharp; TSB: Tryptic Soy Broth; RT: room temperature.

**Table 6:** Microbial inactivation effects of US in different foods.

Treatment	Food Sample	Microorganism	Inactivation (log*N*_0_/*N*)	Condition	Reference
US	Lettuce	*Escherichia coli*	>2	37 kHz/30 W/L	[238]
		*Salmonella* Enteritidis			
	Strawberry	*Escherichia coli*	3.04		
		*Listeria innocua*	6.12		
		*Salmonella* Enteritidis	5.52		
		*Staphylococcus aureus*	2.41		
US	Saline solution and ultrahigh-temperature milk	*Escherichia coli*	>99%	20 kHz/750 W	[239]
		*Saccharomyces cerevisiae*			
US	Saline solution	*Lactobacillus acidophilus*	72%		[240]
	UHT milk		84%		
US	Iranian ultrafiltered feta-type cheese	*Escherichia coli* O157:H7	4.28	60 kHz	[241]
		*Staphylococcus aureus*	1.95		
		*Penicillium chrysogenum*	1.11		
		*Clostridium sporogenes*	2.17		
US	Oyster (Crassostrea gigas)	*Vibrio parahaemolyticus*	3.13	7.5 W/mL/12.5 min	[242]
US	Camel milk	Total bacterial concentration	4.2	160 W	[243]
US	Cabbage	*Listeria monocytogenes* biofilm	4.09 ± 0.12	37 kHz/1550 W	[135]
	Lettuce		3.85 ± 0.12		
HIUS	Chocolate milk	Aerobic mesophilic microorganisms	3.56 ± 0.02	3.0 kJ/cm^3^	[244]
HIUS	Liquid Whole Eggs	*Salmonella* Enteritidis	1.4	20 khz/5 or 10 min	[239]
HIUS	Peanut milk	Yeast and mold	0.9	400 W	[245]
US + plasma	D.I. water	*Escherichia coli*	6	AC (13 kV/60 Hz) US (140 W/47 kHz)	[144]
		Yeast			
US + mild heat	Ultrahigh-temperature milk	*Listeria monocytogenes*	5	20 kHz/150 W/118 W/cm^2^/57 °C	[246]
	Pasteurized apple cider	*Escherichia coli* O157:H7	6		[247]
US + mild Temperatures	Fresh Carrot Juice	*Escherichia coli*	>5	24 KHz/37.87 W/cm^2^/58 °C/2 min	[248]
US + mild Temperatures	Apple cider	*Escherichia coli* K12	5	3W/mL/59 °C/3.8 min	[249]
US + cinnamon essential oil	Low-fat milk	*Listeria monocytogenes*	4.3	24 kHz/400 W/15 min	[250]
		*Salmonella* Typhimurium	2.7		
	High-fat milk	*Listeria monocytogenes*	4.5		[251]
		*Salmonella* Typhimurium	3.8		
US + nisin + oregano	Lettuce	*Escherichia coli* O157:H7	3.43	771.2 IU/g nisin/0.185% v/v oregano/14.65 min	[252]
		*Listeria monocytogenes*	9.20		
US + aqueous chlorine dioxide	Alfalfa	*Salmonella* Enteritidis	1.94 ± 0.42	26 kHz/90 mm/200 W	[253]
		*Escherchia coli*	2.62 ± 0.02		
	Mung bean sprouts	*Salmonella* Enteritidis	2.06 ± 0.23		
		*Escherchia coli*	2.08 ± 0.02		
Low-frequency US + peracetic acid + ascorbic acid	Cherry tomato	*Escherichia coli* O157:H7	0.7–0.9	25 kHz US/1% AA/80 ppm PAA	[238]
		*Salmonella* Typhimurium	0.6–0.8		
		Aerobic mesophilic microorganisms	0.7–1.0		
		Molds and yeasts	0.5–1.0		
US + sodium hypochlorite	Kiwifruit	Aerobic mesophilic microorganisms	3.48	368 W/cm^2^/25 °C/30 ppm NaOCl/8 min	[254]
		Molds and yeasts	2.32		
US + blue light	Salmon	*Vibrio parahaemolyticus*	98.81%	216 J/cm^2^/15 min	[240]
US + lactic acid, acetic acid	Spinach leaves	*Escherichia coli* biofilm	2.86–6.03	35 kHz/380 W/100% power	[255]
	Polystyrene surfaces		6.21	40 kHz/360 W/50 °C/5 min	[256]

HIUS: High-intensity US.

**Table 7:** Comparison of the pasteurization effect and VBNC formation potential of non-thermal technologies.

Non-Thermal Technologies	Advantages	Disadvantages	VBNC Formation Potential
HPP	Good pasteurization effect on various microorganisms; pasteurized with packaged products; has been commercially applied; minimal changes to physicochemical properties of food	High equipment and maintenance costs	VBNC bacteria were formed, which could resuscitate during subsequent storage; bacterial pressure resistance was positively correlated with resuscitable VBNC populations.
PEF	Good pasteurization effect on various microorganisms; short processing time; has been commercially applied; minimal changes to physicochemical properties of food	Limited antimicrobial effect in solid foods	Research results are inconsistent on VBNC formation induced by PEFs.
CAP	Good pasteurization effect on various microorganisms; good effect for surface disinfection; PAW enhances treatment uniformity	Highly limited in the pasteurization effect while working with thick, large, and rough materials; expensive and complicated equipment; generation of ROS and RNS may affect food qualities	VBNC state was formed and VBNCs would resuscitate. VBNC pathogens still maintained pathogenicity.
HPCD	Good pasteurization effect on various microorganisms	The utilization of CO_2_ may cause acidification of products.	VBNC state was formed and VBNCs would resuscitate. VBNC pathogens retained reduced pathogenicity.
US	Good pasteurization effect on various microorganisms	Localized extreme temperatures generate free radicals, and affect food qualities; generates significant operational noise	VBNC state was formed and VBNC pathogens remained virulent. US combined with other pasteurization methods can reduce VBNC population

## Data Availability

No new data were created or analyzed in this study. Data sharing is not applicable to this article.

## Abbreviations

The following abbreviations are used in this manuscript:

HPP	High pressure processing
PEF	Pulsed electric field
CAP	Cold atmospheric plasma
HPCD	High pressure carbon dioxide
US	Ultrasound
VBNC	Viable but nonculturable
